# Genotypic Differences in Phosphorus Efficiency and the Performance of Physiological Characteristics in Response to Low Phosphorus Stress of Soybean in Southwest of China

**DOI:** 10.3389/fpls.2016.01776

**Published:** 2016-11-24

**Authors:** Tao Zhou, Yongli Du, Shoaib Ahmed, Ting Liu, Menglu Ren, Weiguo Liu, Wenyu Yang

**Affiliations:** ^1^College of Agronomy, Sichuan Agricultural UniversityChengdu, China; ^2^Key Laboratory of Crop Ecophysiology and Farming System in Southwest, Ministry of AgricultureChengdu, China

**Keywords:** *Glycine max*, phosphorus, yield, root morphology, organic acids, acid phosphatase

## Abstract

Southwest of China is one of the major soybean (*Glycine max* L.) production regions in China with low availability of soil phosphorus (P). Whereas little information is available on P-efficient soybean genotypes in this region, even though using P-efficient soybean genotypes is a sustainable P management strategy for enhancing yield and P use efficiency. To assess the genetic variation on P use efficiency, 274 soybean genotypes were employed to compare the yields and P acquisition potentials in the field. Additionally, 10 representational genotypes (5 P-efficient genotypes and 5 P-inefficient genotypes) were grown in hydroponic media containing low P treatment (0.05 mM L^−1^) and high P treatment (0.25 mM L^−1^) to further investigate P assimilation characteristics and the related mechanisms of P-efficient soybean genotypes. In the field trial, the models described the relationships between yield and seed P concentration (*R*^2^ = 0.85), shoot P accumulation (*R*^2^ = 0.84), HI (*R*^2^ = 0.82) well. The yield, seed P concentration and shoot P accumulation ranged from 5.5 to 36.0 g plant^−1^, from 0.045 to 0.93% and from 0.065 to 0.278 mg plant^−1^, respectively. In the hydroponic trial, P-efficient genotypes under low P treatment showed significantly better plant growth, P accumulation and root: shoot ratio than P-inefficient genotypes. Simultaneously, total root length, specific root length, root surface area and root volume of P-efficient were significantly greater than P-inefficient under low P treatment. Higher rate of organic acid exudation and acid phosphatase activities was observed in the P-efficient soybean genotypes under low P condition when compared to the P-inefficient soybean genotypes. It indicated that significant genetic variation for P use efficiency existed in this region, and the P-efficient soybean genotypes, especially E311 and E141, demonstrated great tolerance to P deficiency, which could be potential materials using in improving production and P use efficiency in low availability of soil P region.

## Introduction

Low phosphorus (P) availability existed in many soils as inherent P deficiency and/ or P strongly bound to soil particles. Crop production generally relies on regular application of P fertilizer, as P is one of the essential nutrients for crop growth. During the period of 1960–2008, the total grain output of China increased 4.4-fold from 110 to 483 million tons (FAO, 2011), which paid a price of 91-fold P increase (Zhang et al., 2012). Generally, only 15–20% of P can be taken up by crops in the season of application (Zhang et al., 2008). The remaining P is only partially available to crops, while most of them are bound to Al/Fe oxides in acidic soils or to Ca carbonate in alkaline soil and accumulate in soil (Li et al., 2011). Thus, although soil total P concentration ranges from 0.2 g P kg^−1^ to 3.0 g P kg^−1^, less than 1% of the total P is available for crop growth (Otani and Ae, 1996). In fact, P fertilizer is derived from mind phosphate rock, which is a finite resource and is slowly depleting (Cordell et al., 2009). Based on this, Chinese government recently encourages the farmers to decrease chemical fertilizer input in soil, conversely excavating soil P biological availability for improving P use efficiency. More and more attentions were paid on the efficient utilization of P resources (Cordell et al., 2009).

Southwest of China located at the upstream of the Yangtze River and almost the arable field on the hilly landscape with the slow economic development. Besides less P input, tremendous soil erosion by intensive cultivation and rainfall result in soil P deficiency in this region (Zhang et al., 2004; Lin et al., 2009). In 1980, the national average for soil Olsen-P was 7.4 mg kg^−1^. In 2006, the increase of soil Olsen P occurred in all agroecological regions of China. The soil Olsen P in the middle-lower Yangtze plain, in the North China Plain and in the South China was 17.5, 20.7, and 25.4 mg kg^−1^, respectively (Li et al., 2011). However, in 2012, the average of soil Olsen P in Sichuan province was only 16.0 mg kg^−1^. Therefore, excavating soil P biological availability for improving productivity and P use efficiency is vital in the Southwest of China as high risk of P leaching and low P input existed in this area.

Utilization of P-efficient crops has been proved as an effective way to improve P use efficiency. Nutrient-efficient plants are defined as plants which could produce higher yields per nutrient applied or absorbed more compared with other plants grown in similar agroecological conditions (Fageria et al., 2008). Plant root plays the dominant role in increasing in soil P bioavailability and plant P acquisition, and the physiological traits of root can adapt in response to P deficiency. A fine root system includes high length, volume, biomass, specific root length, which is benefit for exploring greater P availability by occupying huge soil volume (Wang et al., 2010). Root exudates, like organic acids and phosphatase enzymes, may also enhance P acquisition by plants. Exudation of organic acids into the rhizosphere successfully increased P availability by mobilizing conservative mineral P and organic P (Po) source, and thus improved plant P acquisition (Dinkelaker et al., 1989). Another typical response of P-efficient genotypes to P deficiency is increasing phosphatase or phytase exudation to mineralize Po (Hayes et al., 2000). Following the development of technology, genetic engineering was used in changing plant physiological and biochemical parameters to improve P use efficiency. Over-expression of organic acids and phytase or acid phosphatase genes lead to significantly increasing in exudation from roots, and therefore enhance plant P acquisition (Koyama et al., 2000; Delhaize et al., 2009; Wasaki et al., 2009). However, most of these results lacked the application in practical production, and improving P use efficiency through genetic improvement is difficult in short time. So the screening of P-efficient plants in the natural conditions is trustworthy and efficacious pathway for pursuing high grain yield and alleviating the conflicts between the depleting P resource and food demand.

Soybean (*Glycine max* L.) not only is an essential source of protein, oil and micronutrients in human and animal diets, but also possesses a pivotal ecological function in cropping system. For instance, improving soil P availability (Xia et al., 2013), nitrogen fixation (Salvagiotti et al., 2008), soil carbon sequestration (Cong et al., 2015) and decreasing soil disease (Gao et al., 2014) for themselves and neighboring. Soybean is widely cultivated all over the world and its cultivation history in China at least 3000 years (Hymowitz, 1970). P-efficient soybean genotypes had been found in South and Northeast of China, and produced a high yield in low P condition (Zhao et al., 2004; Pan et al., 2008). However, soybean is a narrow-adaption crop. Little information was found about P-efficient soybean genotypes in southwest of China, even though where is one of the four major soybean production region in China. Maize/soybean relay strip intercropping system is a dominant cropping system in southwest of China, which makes a weighty contribution in improving crop productions and resources utilization efficiency (Liu et al., 2015). However, soybean in this system undergoes serious shading stress by maize during the common growth stage, which results decreases in yield compared to sole soybean (Yang et al., 2014). P-efficient crops showed more tolerant to low light and P deficiency condition (Wissuwa et al., 2005). Therefore, P-efficient soybean genotypes would be potential materials in improving production and P use efficiency in low light and/ or low P availability agricultural region.

Modern varieties were selected under high input conditions on breeding stations, which may not have the capable of high nutrient use efficiency, because genes controlling traits of benefit under lower soil fertility were lost as they conveyed no advantage under very high soil fertility (Wissuwa et al., 2009). Traditional genotypes showed higher P use efficiency compared with modern varieties when grown in an unfertilized and highly P-fixing soil (Wissuwa and Ae, 2001). In present study, 274 soybean genotypes were collected from Southwest of China with low availability of soil P (Li et al., 2011), and most of these soybean genotypes are traditionally. The objectives of this study were to screening P-efficient soybean genotypes and ascertaining the factors leading to the differences between P-efficient and P-inefficient soybean genotypes. Thus, yield, characteristics of P accumulation, root morphology, activities of Apase and organic acid exudation rate of the P-efficient and P-inefficient soybean genotypes were investigated.

## Materials and methods

### Plant material

**Figure 1:**
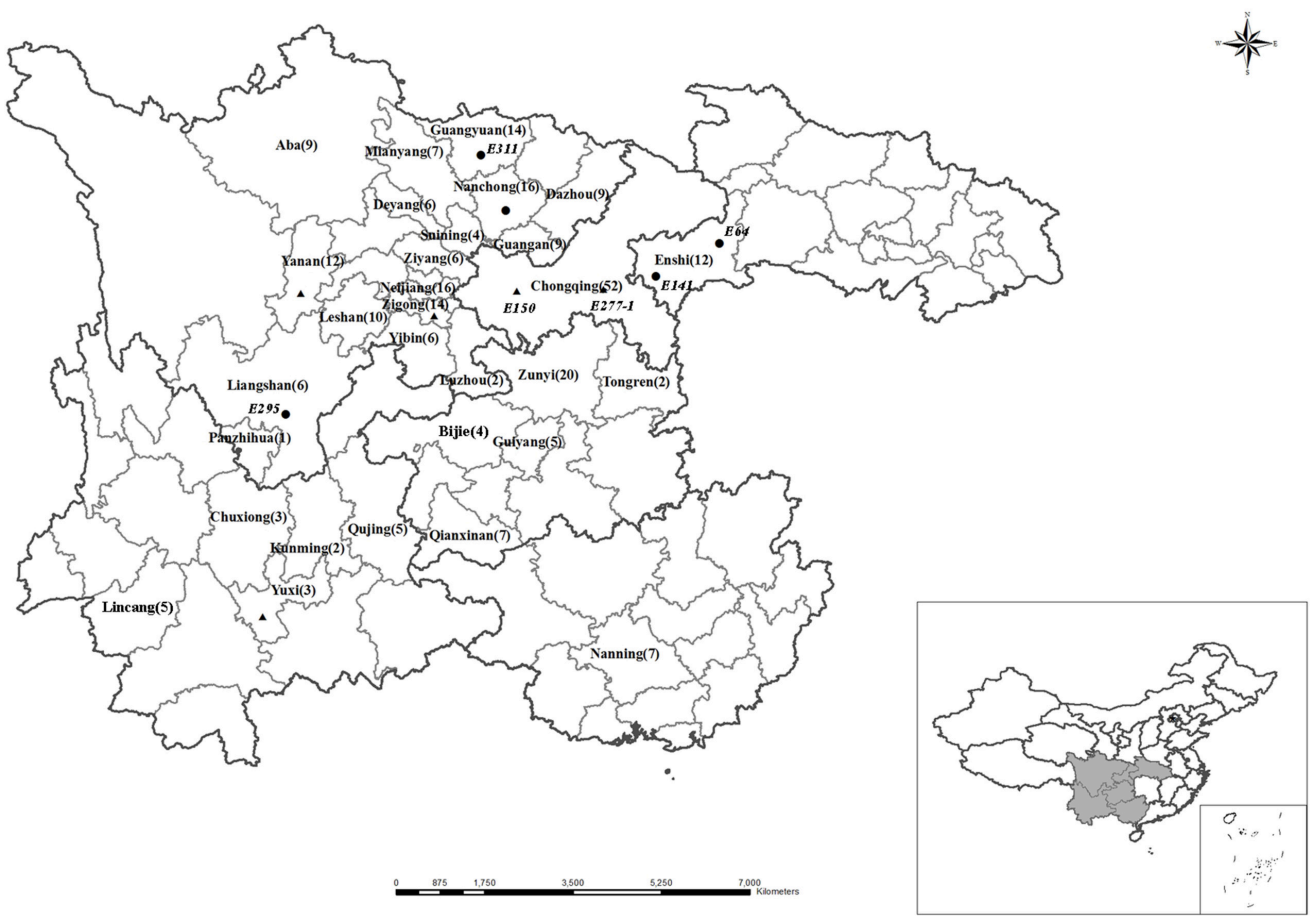
**Map of the Southwest of China (include the province of Sichuan, Yunnan, Guizhou, Chongqing, Hubei, Guangxi) showing the sampling sites**. The numbers in the parentheses represents numbers of soybean genotypes collected from the same city. *Circularity*, P-efficient soybean genotypes sampling sites, *triangle*, P-inefficient soybean genotypes sampling sites in the hydroponic experiment respectively. ☆, Beijing, the capital of China. The position of Southwest of China is shown in the inset. The two maps were created using DIVA-GIS 10.0 software, and the *Circularity, Triangle* sites were added according to GPS records.

Two hundred and seventy-four soybean genotypes (*Glycine max* L.) (Table S1) were collected from Sichuan Province, Yunnan Province, Guizhou Province, Hubei Province, Chongqin City, and Guangxi Zhuang Autonomous Region in the southwest of China (Figure 1). Most of these soybeans are traditional genotype and the gene pool was not domesticated by artificial direct or indirect purpose.

### Field experiment

The field experiment was conducted in 2015 at the experimental station of Sichuan Agricultural University in Ya'an, Sichuan Province, China (29°58′N, 102°58′E) with an altitude of 600 m above sea level. Annual mean temperature is 15.4°C with a maximum and minimum temperature of 25.4° and 6.1°C, respectively. The frost-free period is 294 days, annual precipitation is 1500 mm and potential evaporation is 838 mm. Annual sunshine is 1019 h and total solar radiation averages is 3750 MJ m^−2^ year^−1^. The experimental soil is classified as Purple soil (Luvic Xerosols).

The 274 soybean genotypes were planted in a randomized block design with 3 replicates. Each block consisted of 274 plots with the dimension of 1.5 × 4.0 m^2^. Every plot consisted of three plant rows, and the rows spacing is 50 cm. Density of soybean was about 11 plants m^−2^. Soybean was sown on early June and harvested on late October in 2015. In the last season, maize had been sown in this field, and the soil properties at the start of this study were pH (water) 6.4, organic matter concentration 30.1 g kg^−1^, total N 1.80 g kg^−1^, available N 110 mg kg^−1^, Olsen-P 17.4 mg kg^−1^, exchangeable K 91 mg kg^−1^, and Cation Exchange Capacity 22.0 cmol kg^−1^ of dry soil in the top 20 cm soil layer. During the growth period, all the plots were well irrigated and weeded manually with no fertilizer input.

Shoot dry matter (DM) of soybean was measured at maturity. 10 plants of soybean were sampled from the middle row of each plot. All samples were dried in an oven at 70°C and then ground for further chemical measurements. The samples were wet-digested with concentrated H_2_SO_4_ and H_2_O_2_ (30%) for P determination following the vanadomolybdate method (Page, 1982). Shoot P uptake was calculated as P concentration multiplying with shoot biomass. The harvest index (HI) was calculated as dividing the yield by the shoot biomass. Grain yield of soybean came from harvesting the remaining parts of the plot after shoot sampling.

The linear- plateau model was used to analyze the relationship between yield and HI. The linear-plateau model is defined by Equations (1) and (2) as:

(1) $$\begin{array}{cc} y=a+bx & if(x)<c \end{array}$$

(2) $$\begin{array}{cc} y=Yp & if(x)\geq c \end{array}$$

where *y* is HI; *a* is the intercept parameter; *b* is the slope parameter; *x* is the yield (g/plant); *c* is the critical yield, which is the interception point of the two linear segments; and *Yp* is the plateau value which is often 90% of the maximum HI. Equation (1) can be interpreted as the region during which the HI responds to yield increasing and Equation (2) to the plateau region where increase of yield does not come from the increase of HI.

### Hydroponic P efficient assays

Based on yield and P accumulation of soybean genotypes in the field trial (Table S2), E14, E64, E141, E295, E311 and D55, E108, E150, E277-1, E283 representing the P-efficient, P-inefficient genotypes were used to evaluate tolerance of P deficiency in hydroponic experiment, respectively. Seeds were surface-sterilized as described by Vincent (1970), placed on sterile Whatman filter paper, and germinated in sterile water in pot filled with quartz sand until taproots were 3-4 cm long. The seedlings were transferred to a 24 liter container (0.58 × 0.28 × 0.15 m^3^) filled with half-strength modified Hoagland solution (0.75 mM K_2_SO_4_, 2 mM Ca (NO_3_)_2_, 0.65 mM MgSO_4_, 0.1 mM KCl, 0.2 mM KH_2_PO_4_, 0.1 mM Fe(III)NaEDTA, 10 uM H_3_BO_3_, 1 uM MnSO_4_, 0.1 uM CuSO_4_, 1 uM ZnSO_4_, 0.09 uM (NH_4_)_6_Mo_7_O_24_), each container with 25 soybean plants. The experiment was designed as a complete randomized with two P treatment levels (0.05 and 0.25 mM P L^−1^) and six replicates. Plants were grown in a greenhouse from 14th February to 16th March in 2016 with an average temperature of 25/20°C (day/night), relative humidity 75%, average daytime photosynthetically active radiation between 800 and 1000 mmol m^−2^ s^−1^ and photoperiod of 14 h day/10 h night. The solution was well aerated and renewed every 5 days and pH maintained at 5.4–5.5 with daily regulation. The plants were harvested at stem elongation stage (30 days after sowing) for testing the physiological and root morphology index below. Plants show higher root secretion rate at stem elongation stage than later in growth period, when the amount of fixed C allocated to roots and rhizosphere (Gregory and Atwell, 1991).

### Organic acids in root exudates

For root exudation collection, three replicates of each genotype were washed absolutely with running tap water followed by distilled water and then placed separately in 100 ml glass test tube filled with deionized water and covered with black plastic to prevent light degradation of exudations. The exudate was collected for 6 h, and the details before high performance liquid chromatography (HPLC) analysis refers the method of Dong et al. (2004). Organic acids were analyzed by HPLC (Agilent 1100, Agilent, USA) after Libert and Franceschi (1987) with modifications (Yu et al., 2002). A Hypsil (Hypsil, Dalian, China) C_18_ column (5 uM, 4.6 × 250 mm) was used as the static phase and the mobile phase was a solution containing 0.5% KH_2_PO_4_ and 0.5 mM tetrabutylammonium hydrogen sulfate (TBA) buffered at pH 2.0 with orthophosphoric acid. The flow rate was 1 ml min^−1^ and detection wavelength was 220 nm.

### Determination of biomass and root morphology

After exudate collection, soybean plants immediately divided into shoots and roots. Shoots were dried in an oven at 70°C until constant weight. Roots were placed in clear plastic bags filled with 50% ethanol and stored in a refrigerator. Washed roots were gently arranged with minimum overlap using tweezers in a plexiglass tray full with water, and scanned using an Epson perfection V700 photo, Japan. Images were analyzed using WinRhizo (Version 2007d, Regent Instrument Inc., Canada) to estimate total root length, root surface area, and root volume. Several images of one root were analyzed in more detail to determine the root morphology. Roots were dried in an oven at 70°C until constant weight after imaging. Specific root (cm g^−1^ DW) length was referring to the method of Pang et al. (2010).

### Analysis of root APase activity

After harvest, fresh roots of three replicates were washed absolutely with running tap water and distilled water, and froze immediately in liquid nitrogen, then stored at −80°C. 0.3 g root tissues were ground with a mortar and pestled with 5 mL of 15 mM 2-morpholinoethanesulfonic acid, monohydrate (MES) buffer (pH 5.5, 0.5 mM CaCl_2_ H2O, 1 mM EDTA). The extracts were centrifuged at 4°C for 20 min at 10,000 rpm to obtain the supernatants which were used for the determination of enzyme activity.

P-nitrophenyl phosphate disodium salt hexahydrate (pNPP) was used as substrate to determinate APase activity. At first, 0.5 ml enzyme extract with a total volume of 4 ml containing 15 mM MES buffer and 10 mM pNPP was incubated at 37°C for 30 min. Subsequently, an equal volume of 0.25 M NaOH was added to terminate reaction immediately. APase activity was measured from the release of p-nitrophenol (pNP) and expressed as pNP μg g^−1^ fresh weight (FW) min^−1^, and pNP was determined spectrophotometrically using a UV spectrophotometer (model UN-2600A, UNICO) at 412 nm relatively to standard solutions (Sharma and Sahi, 2005).

### Analysis of tissue P concentration

P concentration of root and shoot was analyzed following the vanadomolybdate method (Page, 1982). Shoot and root P accumulation was calculated by multiplying P concentration with the DW, respectively.

The ratio of root P accumulation: shoot P accumulation is a typical index for plant response to P deficiency. Generally, plant response to P deficiency is the increase in root: shoot ratio, which might be due to preferential assimilate P distribution to the roots (Vance et al., 2003).

### Statistical analysis

Data from 3 replicates were sorted out by Excel (Microsoft) software packages. Regression equations were developed for the relationships between yield and seeds P concentration, shoot P uptake, HI. The liner-plateau model was analyzed by the SAS 9.1.3 software (SAS Institute Inc., USA). The liner model was analyzed by the SPSS 19.0 software. Significant difference of shoot, root dry matter and P accumulation, root length, root surface area, root volume, specific root length, activities of APase and organic acid exudation rate between soybean genotypes and P treatments were analyzed by analysis of variance (ANOVA) and mean values were compared by least significant difference (LSD) multiple comparison using the SPSS19.0 software (SPSS Institute Inc., USA).

## Results

### Field study

#### Grain yield, dry matter accumulation and P acquisition variable in soybean genotypes

**Figure 2:**
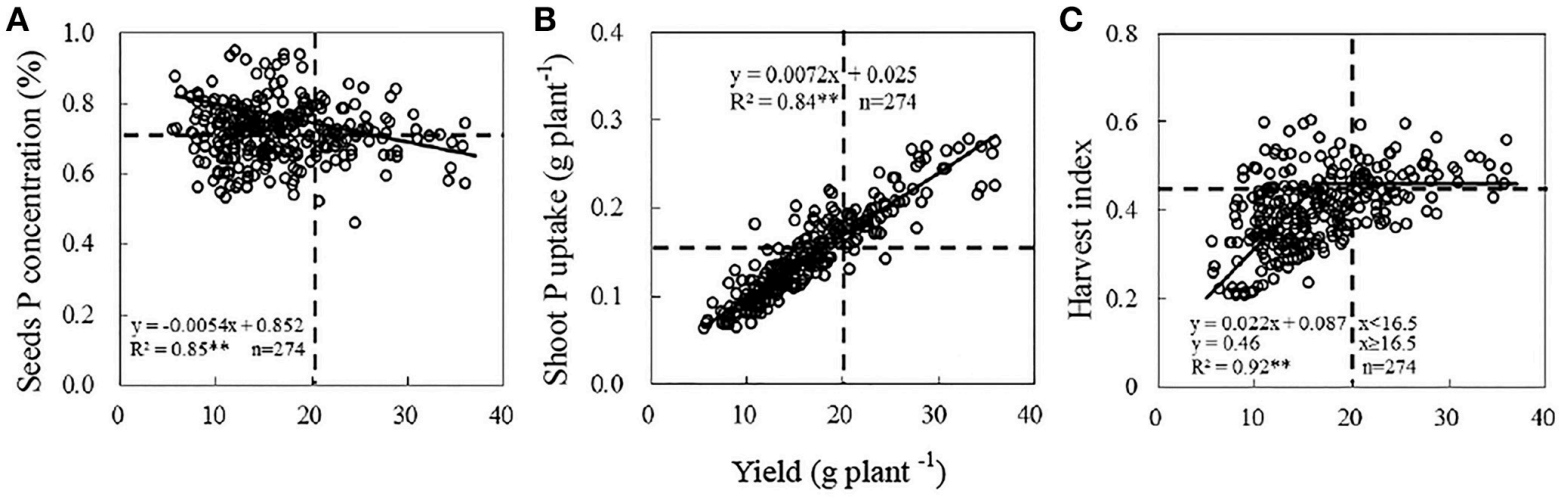
**Relationship between soybean yield and seeds P concentration (A)**, shoot P uptake **(B)**, HI **(C)**. Date are mean of three replicates. The horizontal dotted line in figures **(A–C)** means soybean seeds P content, shoot P uptake and HI among the genotypes, respectively. The vertical dotted lines in figures **(A–C)** were representing the soybean yield of *Nandou 12* in Sichuan province (20 g plant^−1^).

Significant variation existed in grain yield among the 274 soybean genotypes in field experiment (Table S2). Average yield of 274 soybean genotypes ranging from 5.6 to 36.0 g plant^−1^ was 16.5 g plant^−1^. Seed P concentration and shoot P accumulation showed significant response to yield. Moreover, the linear-plateau model described the relationship between yield and HI well (*R*^2^ = 0.82) (Figure 2).

To further compare the partitions of seed P concentration, shoot P accumulation, HI among different soybean varieties, pooled grain yields were divided into two yield categories: <20 g plant^−1^ (low yield, LY) (the number of soybean genotypes, *n* = 206, mean yield: 13.6 g plant^−1^), ≥20 g plant^−1^ (high yield, HY) (the number of soybean genotypes, *n* = 68, mean yield: 25.3 g plant^−1^). 20 g plant^−1^ is the yield of *Nandou 12*, which is Sichuanses summer soybean cultivar with the largest acreage, and the line is also the standard for choose HY soybean genotypes. Pooled seed P concentration, shoot P uptake and HI were divided into two groups below or above the mean line (Figure 2). We delimit the P-efficient soybean genotypes with high yield, shoot P accumulation, HI and seed P concentration simultaneously. On the contrary, the P-inefficient genotypes may be with low yield, shoot P accumulation, HI and seed P concentration. There are 27, 66, and 39 soybean genotypes with high (above the mean line) seed P concentration, shoot P accumulation and HI in HY category, respectively (Figure 2). However, only 16 genotypes possessed high yield, seed P concentration, shoot P accumulation and HI simultaneously. Otherwise, 99, 161, and 171 soybean genotypes possessed low (below the mean line) seed P concentration, shoot P accumulation and HI in LY category, respectively. Considering some genotypes from same site may be with same genetic background, so represent (P-efficient, P-inefficient) soybean genotypes with a large distance scale between each other were chosen. E14, E64, E141, E295, and E311 were chosen to represent the P-efficient soybean genotypes and D55, E108, E150, E277-1 and E283 were chosen to represent the P-inefficient soybean genotypes.

### Hydroponic study

#### Effect of P supply on dry matter accumulation

**Figure 3:**
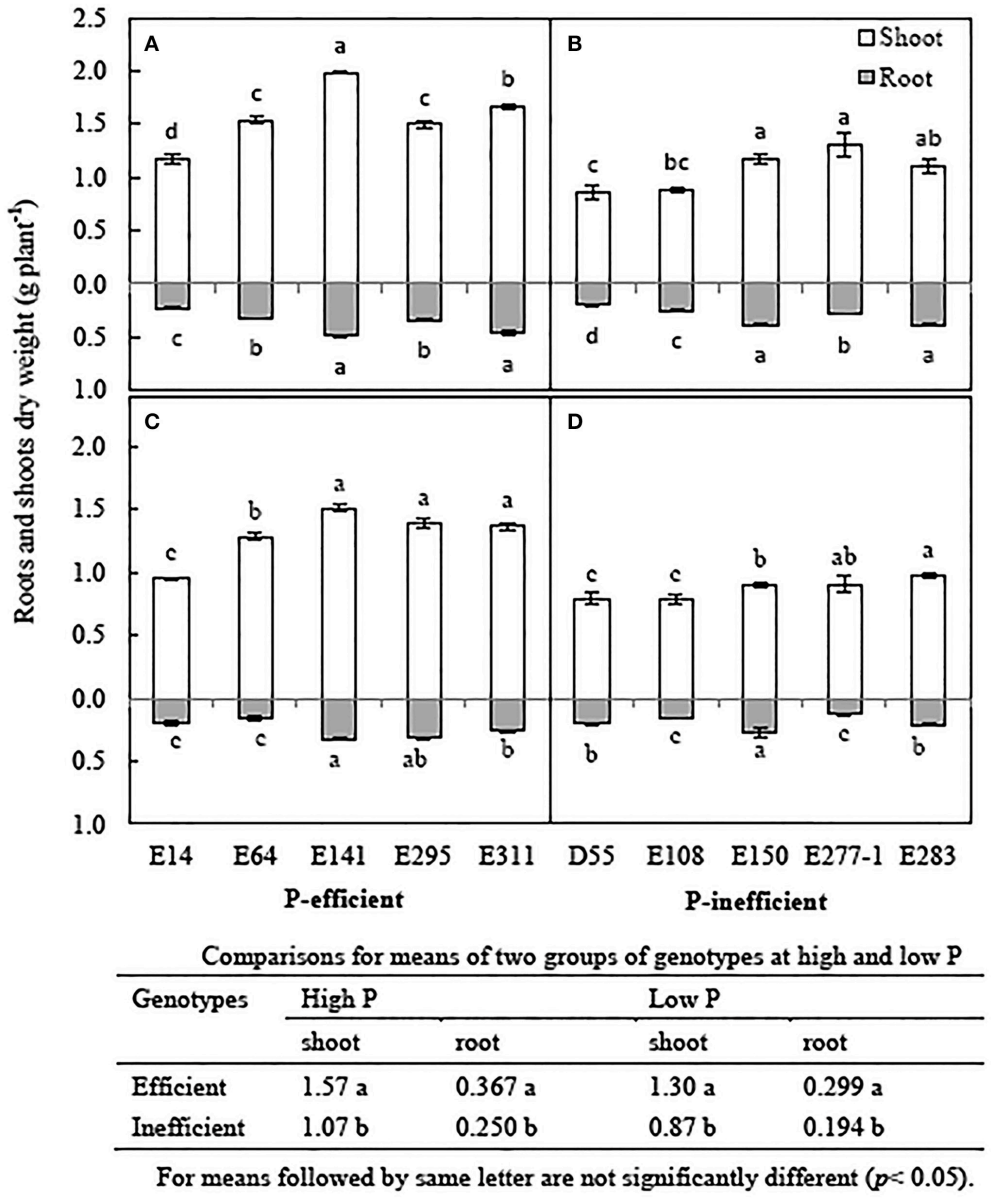
**Root and shoot dry weight of soybean genotypes, grown in high and low P conditions in greenhouse**. Data are mean of three replicates ± SE. **(A)** Biomass of P-efficient soybean genotypes in high P condition. **(B)** Biomass of P-inefficient soybean genotypes in high P condition. **(C)** Biomass of P-efficient soybean genotypes in low P condition. **(D)** Biomass of P-efficient soybean genotypes in low P condition. Different letters on each column of shoot or root are significantly difference at the 5% level by LSD among soybean genotypes.

The biomass of soybean genotypes reduced by 6.1~35.1% in low P treatment compared to in high P treatment (Figure 3). Low P level reduced plant biomass by 19.8% averaged the 10 soybean genotypes, but affected the two groups of phenotypes differently. The average shoot biomass, root biomass of P-efficient soybean genotypes was 49.4 and 54.1% higher than that of the P-inefficient genotypes in low P treatment, respectively (Figures 3C,D). Significant difference for shoot and/ or root was also observed among the genotypes in P-efficient and P-inefficient group. The root and shoot biomass of E141, E295, and E311 significantly higher than E14 and E64 in low P condition (Figure 3C). The root biomass of E150 is the highest in P-inefficient group in low P level (Figure 3D).

#### P accumulation and distribution

**Figure 4:**
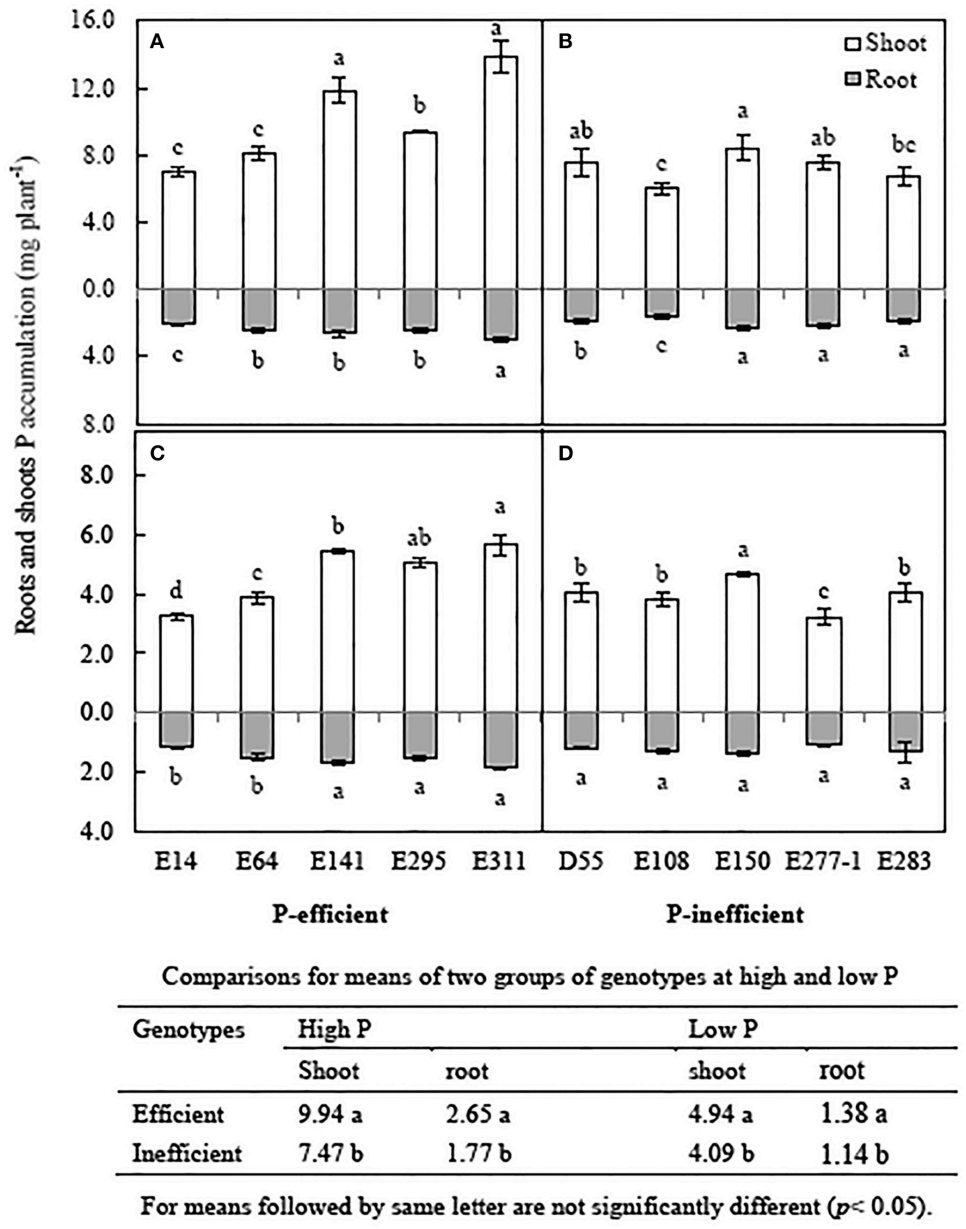
**Root and shoot P accumulation of soybean genotypes, grown in high and low P conditions in greenhouse**. Data are mean of three replicates ± SE. **(A)** P accumulation of P-efficient soybean genotypes in high P condition. **(B)** P accumulation of P-inefficient soybean genotypes in high P condition. **(C)** P accumulation of P-efficient soybean genotypes in low P condition. **(D)** P accumulation of P-efficient soybean genotypes in low P condition. Different letters on each column of shoot or root are significantly difference at the 5% level by LSD among soybean genotypes.

Low P level reduced P accumulation of soybean genotypes by 32.3~55.8% compared to in high P condition. Averaged P accumulation of 10 soybean genotypes reduced by 46.5% in low P level compare to in high P level (Figure 4). The average shoot P accumulation and root P accumulation of 5 P-efficient soybean genotypes was 17.3 and 22.2% higher than that of P-inefficient genotypes in low P condition, respectively (Figures 4C,D). P accumulation of E311 were 7.5 and 16.9 mg plant^−1^ in low and high P level respectively, and were 1.4, 1.7 and 1.6, 1.9-folds greater than that of the E64 and E14 grown in media supplied with low and high P concentration, respectively (Figures 4A,C). Shoot P accumulation of E150 was highest among D55, E108, E277-1 and E283, but root P accumulation of the 5 soybean genotypes have no obviously difference (Figures 4B,D).

As shown in Table 1, the ratio of root P accumulation: shoot P accumulation of P-efficient soybean genotypes in low P condition was much high than in high P condition. However, the ratio of the P-inefficient genotypes in low and high P conditions showed narrow difference. For instance, the ratios of E141 and E311 in low P condition were 30.8%, 32.9% and 30.0%, 50.8% higher than in high P level, respectively. But the ratio of E150 in low P condition was 30.0%, and higher than in high P level 9.7%. Low P condition increased the root: shoot ratios of soybean genotypes, but P-efficient soybean genotypes preferentially assimilate P distributed in root.

**Table 1:** **P distribution between root and shoot of soybean genotypes grown in low and high P conditions in greenhouse**.

**Efficient genotypes**	**Root P accumulation: shoot P accumulation ratio (%)**				**Inefficient genotypes**
	**High P**	**Low P**	**High P**	**Low P**
E14	29.8	35.4	25.6	29.6	D55
E64	30.5	39.0	26.8	34.4	E108
E141	22.3	30.8	27.4	30.0	E150
E295	26.6	29.9	28.1	33.2	E277-1
E311	21.8	32.9	27.5	32.5	E283

*Data are mean of three replicates. Ten genotypes were separated into two groups (P-efficient group included E14, E64, E141, E295, E311, P-inefficient group included D55, E108, E150, E277-1, E283). The ratio of root P accumulation: shoot P accumulation was counted by root P accumulation divided by shoot P accumulation*.

#### Root morphology

Low P reduced root length, root surface area and root volume of soybean genotypes compared with in high P level (Table 2). However, P-efficient soybean genotypes had greater root length, surface area and root volume than P-inefficient genotypes in low and high P condition (Table 2). E141 possessed the highest root length, surface area and root volume in high P level, but those root morphology index decreased by 47.8, 51.2, and 56.4% in the low P level, respectively. On the contrary, root length, root surface area and root volume of E311 in low P level just decreased by 17.4, 16.4, and 2.3% compared with in high P level, respectively (Table 2). Low P level reduced specific root length of P-inefficient soybean genotypes compared with in high P level, except for E283. But, E64, E141, and E311 reached a higher specific root length (9837, 9381, and 10063 m g^−1^ DW, respectively) in low P level than in high P level (Table 2).

**Table 2:** **Root morphology of soybean genotypes grown in low and high P conditions in greenhouse**.

**Genotypes**	**Root length (cm plant**^**−1**^**)**		**Root surface area (cm**^**2**^ **plant**^**−1**^**)**		**Root volume (cm**^**3**^ **plant**^**−1**^**)**		**Specific root length (cmg**^**−1**^ **plant**^**−1**^**)**	
	**High**	**Low**	**High**	**Low**	**High**	**Low**	**High**	**Low**
**P-EFFICIENT**								
E14	1926 c^*^	546 d	279 c^*^	112 c	3.26 c^*^	1.31 c	8579 a^*^	6794 c
E64	3020 b^*^	1523 c	379 b^*^	187 bc	3.81 bc^*^	1.81 c	9350 a	9837 b
E141	3985 a^*^	2078 b	514 a^*^	251 b	5.44 a^*^	2.37 b	8147 a	9381 b^*^
E295	2313 c^*^	1354 c	343 c^*^	246 b	4.01 b^*^	2.32 b	6853 b^*^	4248 d
E311	3117 b^*^	2575 a	469 ab^*^	392 a	4.21 b	4.12 a	6772 b	10063 a^*^
**P-INEFFICIENT**								
D55	1196 d^*^	568 c	141 c^*^	75 c	1.36 c^*^	0.79 c	6057 bc^*^	2805 c
E108	1392 c^*^	925 b	196 b^*^	133 b	2.17 b^*^	1.49 b	5618 c	5041 b
E150	2451 a^*^	1584 a	316 a^*^	219 a	3.48 a^*^	2.20 a	6400 b^*^	5801 ab
E277-1	1342 c^*^	1147 b	221 b^*^	149 b	1.56 c	1.51 b	7725 a^*^	5058 b
E283	1660 b^*^	1386 a	219 b^*^	165 b	2.29 b^*^	1.55 b	5325 c	6556 a^*^
**AVERAGE**								
Efficient	2872 A^*^	1615 A	397 A^*^	238 A	4.15 A^*^	2.39 A	7940 A	8065 A
Inefficient	1608 B^*^	1122 B	219 B^*^	148 B	2.17 B^*^	1.51 B	6225 B^*^	5052 B

*Data are mean of three replicates. Values followed by the same small letters in each column are not significantly different among different soybean genotypes in one group at the 5% level by LSD. Capital letters in each column are significantly different (p <0.05) between the average of efficient and inefficient group*.

* *Indicated significantly different (p <0.05) between the two P level*.

#### Activities of APase

**Figure 5:**
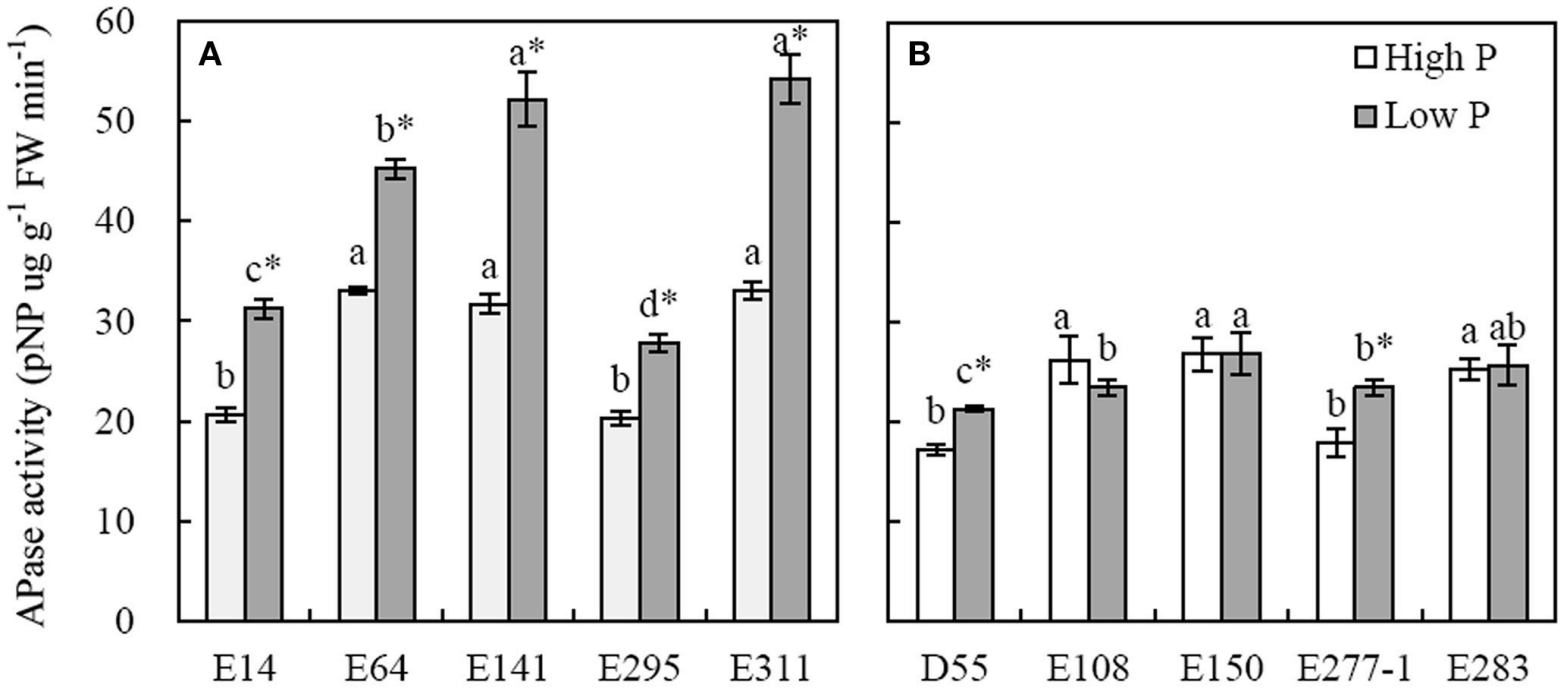
**Activities of APase in roots of soybean genotypes grown in high and low P conditions in greenhouse**. Data are mean of three replicates ± SE. **(A)** Root APase activities of P-efficient soybean genotypes. **(B)** Root APase activities of P-inefficient soybean genotypes. Different letters on each column are significantly different among soybean genotypes at the 5% level by LSD. *Indicates significantly different (*p* <0.05) between high and low P level.

APase activity of root significantly increased in low P level compared to in high P condition (Figure 5). The root APase activities of P-efficient soybean genotypes ranged from 27.8 to 54.21 pNP ug (g FW)^−1^ min^−1^, and the average was 42.1 pNP ug (g FW)^−1^ min^−1^ in low P level (Figure 5A). The corresponding data of P-inefficient soybean genotypes ranging from 20.29 to 33.09 pNP ug (g FW)^−1^ min^−1^ was averaged by 24.1 pNP ug (g FW)^−1^ min^−1^ in low P level (Figure 5B). The maximum APase activities of E311 and E141 reached 52.15 and 54.21 pNP ug (g FW)^−1^ min^−1^ in low P level, respectively, and were 1.2~2.3-fold higher than others (Figure 5A). D55 and E277-1 showed higher APase activities in low P condition than the E108, E150, and E283 in high P level (Figure 5B).

#### Organic acid exudation

Root exudation of malate was greatly motivated in both P-efficient and P-inefficient soybean genotypes by P deficiency (Table 3). The maximum malate exudation rate of 114.6 and 134.8 mg plant^−1^ h^−1^ were found in E311 and E141 in low P level, respectively. Citrate and oxalate exudation of P-efficient and P-inefficient soybean genotypes increased in low P condition. P-efficient soybean genotypes sustained a higher rate of citrate and oxalate efflux than P-inefficient genotypes in low P level. The tartrate efflux was activated by P deficiency and hereditary character. E141 and E311 maintained a high rate of malate, citrate, tartrate, and oxalate excretion, especially in P deficiency condition. Indicating that organic acid exudation by soybean roots was in response to P starvation and genotypes. E150 maintained a high rate of malate, citrate and oxalate excretion than the other four soybean P-efficient genotypes in low P condition. On the contrary, E277-1 and E283 maintained a high rate of tartaric excretion than D55, E108 and E150 in low P level (Table 3).

**Table 3:** **Organic acids exudation rate of soybean genotypes grown in low and high P conditions in greenhouse (mg plant^−1^ h^−1^)**.

**Genotypes**	**Malic acid**		**Citric acid**		**Tartaric acid**		**Oxalic acid**	
	**High**	**Low**	**High**	**Low**	**High**	**Low**	**High**	**Low**
**P-EFFICIENT**								
E14	18.8 c	26.3 e^*^	2.6 c	6.7 bc^*^	2.3 c	9.4 cd^*^	0.3 c	2.6 b^*^
E64	20.8 c	81.2 c^*^	3.4 b	13.0 a^*^	11.2 b	12.2 c	0.5 bc	1.5 c^*^
E141	33.0 b	134.8 a^*^	4.2 a	13.8 a^*^	19.5 a	36.4 a^*^	0.8 b	7.1 a^*^
E295	24.8 c	37.5 d^*^	2.6 c	4.8 c	4.3 c	7.9 d^*^	0.8 b	1.9 c^*^
E311	77.3 a	114.6 b^*^	3.7 b	9.4 b^*^	3.2 c	23.4 b^*^	2.4 a	3.3 b^*^
**P-INEFFICIENT**								
D55	7.3 b	7.2 b	2.7 b	2.0 c	3.4 b	3.4 b	1.5 a^*^	0.7 c
E108	3.8 c	10.4 b^*^	2.4 b	2.8 c	3.9 b	4.7 b	0.5 b	0.9 c
E150	18.6 a	52.0 a^*^	2.8 b	13.2 a^*^	7.2 a	5.5 b	0.5 b	2.3 a^*^
E277-1	12.5 b	51.6 a^*^	1.9 c	8.3 b^*^	6.3 a	9.5 a^*^	0.5 b	1.4 b^*^
E283	24.4 a	54.6 a^*^	3.5 a	7.6 b^*^	3.9 b	7.0 a^*^	1.2 a	1.4 b
**AVERAGE**								
Efficient	34.9 A	78.9 A^*^	3.3 A	9.5 A^*^	8.1 A	17.9 A^*^	1.0 A	3.3 A^*^
Inefficient	13.3 B	35.2 B^*^	2.6 B	6.8 B^*^	4.9 B	6.0 B	0.8 B	1.3 B^*^

*Data are mean of three replicates. Values followed by the same small letters in each column are not significantly different among different soybean genotypes in one group at the 5% level by LSD. Capital letters in each column are significantly different (p <0.05) between the average of efficient and inefficient group*.

* *Indicated significantly different (p <0.05) between the two P level*.

## Discussion

### Genetic variations existed for grain yield and P accumulation in the Southwest of China

To screen P-efficient soybean genotypes for achieving high yield and improving P use efficiency, 274 soybean genotypes were collected from Southwest of China (Figure 1). Besides low availability of soil P, low P use efficiency partly resulted from the behavior of fertilizer P application by farmers, who often input more P than crops need (Vitousek et al., 2009). The remaining P accumulation in the soil is easily eroded with soil by rainfall (Zhang et al., 2012). To sustainably improve crop production and fertilizer P used efficiency through exploiting the biological P potential in the soil, employing the P-efficient genotypes is an efficacious strategy (Li et al., 2011). In this study, grain yield, shoot P accumulation, seed P concentration and harvest index (HI) of 274 soybean genotypes were analyzed in Purple soil with a suitable soil P concentration (initial Olsen-P concentration was 17.4 mg kg^−1^). The results showed that seed P concentration was diluted and shoot P accumulation improved following increasing yield (Figure 2). The 274 soybean genotypes were originated from a large span area with low availability of soil P (Figure 1), which differed substantially in grain yield and P accumulation potentials (Figure 2). The yield of 274 soybean genotypes ranged from 5.5 to 36.0 g plant^−1^. The seed P concentration and shoot P accumulation ranged from 0.045 to 0.93% and 0.065 mg plant^−1^ to 0.278 mg plant^−1^, respectably (Figure 2). It suggested that genetic variations existing in the 274 soybean genotypes, and which provide a potential for assessment P-efficient soybean genotypes (Pan et al., 2008). Such substantial genotypic variation in response to different P use efficiency was also detected for a considerable number of soybean genotypes in South and Northeast of China (Zhao et al., 2004; Pan et al., 2008). In the field study, based on the stand enumerated before 5 P-efficient and 5 P-inefficient soybean genotypes were chosen (Figure 2). Obviously, the evidence mentioned above to proving the soybean genotypes with different P use efficiency is not enough, much more work should be provided to support the results as root physiological, chemical characteristics. Hydroponic study is a fine pathway to check plant root characteristics as it is pellucid and efficient by short plant growth period.

### Biomass and P accumulation of soybean genotypes

Crop P efficiency was defined as the ability to produce biomass or yield under certain available P supply conditions (Wissuwa et al., 2009), and further explained the capability of P uptake from the media and tolerance in P insufficient conditions (Wissuwa and Ae, 2001; Wissuwa et al., 2005; Wang et al., 2010). In the hydroponic experiment, P-efficient genotypes exhibited better adaptability and tolerance than the P-inefficient genotypes grown in low P solution (Figure 3C). Especially E141 and E311, showed significantly greater biomass than that of P-inefficient genotypes in low P level (Figure 3). These results corresponded well with previous studies which showed that the P-efficient plant genotypes demonstrated greater biomass compared to the P-inefficient when grown in low P condition. Some scholars had reported that P-efficient plants of soybean (Zhao et al., 2004), rice (*Oryza sativa* L.) (Wissuwa and Ae, 2001; Mori et al., 2016), maize (*Zea mays* L.) (Zhang, 2012), and *Brassica napus* (Zhang et al., 2009) showed significantly biomass ascendancy compared with P-inefficient plants in P shortage conditions.

Early studies suggested that P-efficient plants preferential distribution P to roots for improving the tolerance to P deficiency and stimulate root growth and P uptake (Wissuwa et al., 2005). In present study, much more P distribution to roots of P-efficient soybean genotypes in low P condition may be an adaptation involved in increasing the tolerance to P deficiency (Table 1). Tolerant plants have been more efficient in P uptake per root size and the additional P then drove further root growth, assuming that low P availability affected root biomass accumulation directly (Wissuwa et al., 2005). In this study, P accumulation of E141 and E311 was higher than the other genotypes in low P solution (Figure 4C). Some scholars reported that P-efficient soybean genotypes are able to obtain sufficient P from acid red soil and alkaline soil under P lack conditions (Zhao et al., 2004; Pan et al., 2008), and other P-efficient plant species also showed positively P accumulation grown in P lack conditions, like maize (Liu et al., 2004), wheat (*Triticum aestivum* L.) (Fageria and Baligar, 1999) and rice (Mori et al., 2016). It indicated that the P-efficient soybean genotypes (especially E141 and E311) demonstrate great capability on P absorption and accumulation potentials.

### Root physiological adaptation concerned in stimulate P assimilation

Root physiological adaptations play important roles in enhancing soil P bioavailability and crop P use efficiency (Shen et al., 2011). These adaptation mechanisms mainly include altering root morphology to enhance P absorption (Wissuwa, 2003), facilitating organic acids exudation into the rhizosphere to increase P availability by mobilizing sparingly soluble mineral P and organic P sources (Johnson et al., 1996), and promoting phosphatase exudation to mineralize Po (Li et al., 2012). Root morphological traits closely linked with P acquisition ability of plants (Pang et al., 2010), and fine root morphology is propitious to maximize P assimilation. P-efficient soybeans showed high parameters of root length, root surface area and root volume than the P-inefficient in present study (Table 2). The specific root length of E311 increased by 48.6% compared with that in high P level (Table 2). It indicated that penurious P availability has negative effects on root morphology, however the less reduction of those root morphology parameters of P-efficient genotypes seems to be the results of excellent tolerance and adaptation. It reported that P-efficient plant genotypes altered root morphology to adapt low P conditions, and then assimilated more P than P-inefficient genotypes (Fita et al., 2011). There was great possibility for plants in improving soil P excavation and utilization by increasing root length, root surface area, root volume and specific root length(Vance et al., 2003), which result in a large total amount of P and dry matter accumulation (Zhang et al., 2013).

Besides root morphology, exudation of APase to improve P bioavailability is another important root physiological adaptation. As we know, exudation of APase by roots increased under P deficiency condition, and plant roots with high APase activities have great potential to utilize soil Po (Hayes et al., 2000; Lung and Lim, 2006). In this study, the results corresponded well with earlier researches that P shortage conditions stimulate the APase activity of soybean genotypes, especially the P-efficient soybean genotypes (Figure 5). Po comprises 30–80% of the total P in most agricultural soils (Dalai, 1977), it can be released through mineralization processes mediated by enzymes activity. The P-efficient plant species generally had high enzymes activity in root extracts when grown in P lack or high Po media. For instance, maize, lupin and chickpea showed high APase activities when grown in P deficiency conditions (Wasaki et al., 2003; Li et al., 2004), *Gulf*, *Marshall Ryegrass* and *P. hydropiper* had greater APase activities when grown in phytate-sufficient media (Sharma and Sahi, 2011; Huang et al., 2012). In our results, the APase activities of E311 and E141 were significantly higher (1.2~2.3-folds) than others in low P level (Figure 5C), suggesting that they demonstrated great capabilities of activation and utilization soil P. Besides, transgenic expression of APase gene in P-efficient genotypes was another path for great dry matter and P accumulation. It reported that over-expressed *AtPAP15* in soybean and *GmPAP4* in *Arabidopsis* significantly increased dry matter and P accumulation compared to the wild type when grown in media with Po (Wang et al., 2009; Kong et al., 2014). Above all, APase activity was a symbol of efficient mineralization and utilization soil P by plants.

Root exudation of organic acids into the rhizosphere had been proposed to improve soil P availability and plant P accumulation (Dinkelaker et al., 1989; Johnson et al., 1996). Exudation of organic acids is also an important root physiological adaptation to P deficiency. A 2-fold increase in exudation of citrate was observed under P starvation in alfalfa (Lipton et al., 1987). Dong et al. (2004) also reported that oxalate and malate exudation of soybean plants was found to markedly increase in response to P deficiency. In present study, low P condition stimulates exudation of malate, citrate, tartrate and oxalate acid, especially in the P-efficient soybean genotypes (Table 3). The average exudation rate of malate, citrate, tartrate and oxalate acid in the P-efficient soybean genotypes increased by 2.24, 1.40, 2.98, and 2.54 folds compared to the P-inefficient genotypes under P starvation, respectively. Particularly, E141 and E311 exhibited high organic acid exudation potentials (Table 3). These results were in agreement with Dong et al. (2004), who reported that a considerable amount of organic acid exudation in P-efficient soybean genotypes contributed to P accumulation under P starvation condition. Adequate evidences demonstrated that root exudation of organic acids into the soil rhizosphere contributed to higher P acquisition. Exudation of malate in P-efficient faba bean enhanced P acquisition in the alkaline soil (Rose et al., 2010). Barley (*Hordeum vulgare* L.) genotypes expressing the *TaALMT1* gene from wheat improved P uptake per unit root length from an acid soil (Delhaize et al., 2009).

## Conclusion

Field study provided evidence that P use efficiency difference exist in 274 soybean genotypes. The hydroponic study suggested that the P-efficient genotypes are more tolerant to P deficiency, and exhibited fine root morphology and physiological adaptations. In conclusion, our results demonstrated that genotypic variation on P use efficiency existed in yield, P accumulation potentials and root physiological characteristics in the Southwest of China by field and hydroponic experiments. We suggested that P-efficient soybean genotypes, like E311 and E141, with fine root morphology, high level of root APase activities and exudation of organic acids rate could be potential materials in breeding and improving the production and P use efficiency in intensive agricultural region with low soil P availability. Interestingly, P-inefficient genotype E150 gained low yield in the field but showed fine root morphology and physiological adaptations in the hydroponic experiment, further studies are needed to probe the reason of this phenomenon.

## Author contributions

TZ, WL, and WY carried out the design of this research work and writing this paper. TZ, YD, and SA carried out the plant cultivation, chemical analysis and statistical analysis of this work. TL and MR participated in experiment management.

### Conflict of interest statement

The authors declare that the research was conducted in the absence of any commercial or financial relationships that could be construed as a potential conflict of interest.
