# Supplementary material for: Genotypic Differences in Phosphorus Efficiency and the Performance of Physiological Characteristics in Response to Low Phosphorus Stress of Soybean in Southwest of China
Source: Front Plant Sci. 2016 Nov 24;7:1776. doi: 10.3389/fpls.2016.01776 (PMC5121124; doi:10.3389/fpls.2016.01776)
Supplement: Supplementary file 1 [file Table1.DOCX]

**Table S1 Origin details of the 274 soybean genotypes used in the present experiment**

| **Genotypes name** | **Sampling sties** | **Genotypes name** | **Sampling sties** |
| --- | --- | --- | --- |
| E237-2 | Aba (9), Sichuan | A26 | Guangyuan (14), Sichuan |
| E237-3 |  | A28 |  |
| E237-5 |  | E233 |  |
| E238 |  | E25 |  |
| E246-2 |  | E288-1 |  |
| E247 |  | E311 |  |
| E248 |  | E312 |  |
| E250 |  | E313 |  |
| E315 |  | E314 |  |
| A46 | Dazhou (9), Sichuan | E317 |  |
| E112 |  | E38 |  |
| E244 |  | E44 |  |
| E244-1 |  | E67 |  |
| E255-1 |  | E68 |  |
| E256 |  | A33 | Leshan (10), Sichaun |
| E257 |  | E129 |  |
| E305 |  | E130 |  |
| E45-1 |  | E133 |  |
| E11 | Deyang (6), Sichuan | E179 |  |
| E12 |  | E28 |  |
| E183 |  | E29 |  |
| E184 |  | E290 |  |
| E336 |  | E316 |  |
| E337 |  | E350 |  |
| A40 | Guangan (9), Sichaun | D9 | Liangshan (6), Sichuan |
| A44 |  | E295 |  |
| A44-1 |  | E296 |  |
| A44-2 |  | E299 |  |
| A45 |  | E47 |  |
| D10 |  | E55 |  |
| E178 |  | A12-1 | Luzhou (2), Sichuan |
| E23 |  | A12-2 |  |
| E42 |  | A9 | Panzhihua (1), Sichuan |
| A17 | Mianyang (7), Sichaun | D24 | Yibin (6), Sichuan |
| E199 |  | E24 |  |
| E205 |  | E7 |  |
| E31 |  | E71 |  |
| E347 |  | E8 |  |
| E351 |  | E9 |  |
| E56 |  |  |  |

**Table S1 continued**

| **Genotypes name** | **Sampling sties** | **Genotypes name** | **Sampling sties** |
| --- | --- | --- | --- |
| A36 | Nanchong (16), Sichuan | A57 | Yaan, continued |
| C103 |  | E161 |  |
| C57 |  | E161-1 |  |
| C68 |  | E162 |  |
| D15 |  | E163 |  |
| D16 |  | E167 |  |
| E14 |  | E212 |  |
| E19 |  | E285 |  |
| E191 |  | E355 |  |
| E192 |  | E53 |  |
| E201 |  | E54 |  |
| E202 |  | A18 | Ziyang (6), Sichuan |
| E302 |  | D18 |  |
| E318 |  | D19 |  |
| E320 |  | D25 |  |
| E352-1 |  | D31 |  |
| A29 | Neijiang (16), Sichaun | D33 |  |
| A29-1 |  | A6 | Zigong (14), Sichuan |
| A30 |  | A8 |  |
| A32 |  | D43 |  |
| D3 |  | D44 |  |
| E106 |  | D48 |  |
| E13 |  | D50 |  |
| E13-1 |  | D51 |  |
| E234-1 |  | D55 |  |
| E234-2 |  | E203-1 |  |
| E26 |  | E204 |  |
| E26-1 |  | E204-1 |  |
| E27 |  | E236 |  |
| E29-1 |  | E72 |  |
| E33 |  | E95-1 |  |
| E33-1 |  | D69 | Chongqing (52) |
| A25 | Suining (4), Sichuan | D28 |  |
| E279-1 |  | D30 |  |
| E280 |  | D70 |  |
| E282 |  | D71 |  |
| A52 | Yaan (12), Sichuan | D72 |  |

**Table S1 continued**

| **Genotypes name** | **Sampling sties** | **Genotypes name** | **Sampling sties** |
| --- | --- | --- | --- |
| D74 | Chongqing continued | E375-5 | Chongqing continued |
| D75 |  | E60 |  |
| D76 |  | E61 |  |
| D77 |  | E65 |  |
| E143 |  | E66 |  |
| E144 |  | E94 |  |
| E145 |  | E115 | Nanning (7), Guangxi |
| E146 |  | E116 |  |
| E147 |  | E117 |  |
| E148 |  | E118 |  |
| E150 |  | E211 |  |
| E152 |  | E300 |  |
| E153 |  | E70 |  |
| E154 |  | E127 | Enshi (12), Hubei |
| E155 |  | E134 |  |
| E156 |  | E135 |  |
| E175-2 |  | E136 |  |
| E176-2 |  | E137 |  |
| E176-5 |  | E138 |  |
| E20 |  | E139 |  |
| E20-1 |  | E140 |  |
| E21 |  | E141 |  |
| E22 |  | E142 |  |
| E235 |  | E63 |  |
| E239 |  | E64 |  |
| E241 |  | E119 | Bijie (4), Guizhou |
| E24-2 |  | E119-1 |  |
| E24-3 |  | E121 |  |
| E260 |  | E193 |  |
| E274 |  | E15 | Guiyang (5), Guizhou |
| E275 |  | E16 |  |
| E277 |  | E17 |  |
| E277-1 |  | E18 |  |
| E304 |  | E99 |  |
| E32 |  | E180 | Tongren (2), Guizhou |
| E322 |  | E180-1 |  |
| E342 |  | E230 | Qianxinan (7), Guizhou |
| E346 |  | E232 |  |
| E36 |  | E276 |  |
| E37 |  | E286 |  |

**Table S1 continued**

| **Genotypes name** | **Sampling sties** | **Genotypes name** | **Sampling sties** |
| --- | --- | --- | --- |
| E325 | Qianxinan continued | 16 | Chuxiong (2), Yunnan |
| E98 |  | 17 |  |
| E98-1 |  | E107 | Kunming (2), Yunnan |
| E122 | Zunyi (20), Guizhou | E107-1 |  |
| E126 |  | 18 | Lincang (5), Yunnan |
| E157 |  | 19 |  |
| E157-1 |  | 23 |  |
| E157-2 |  | 24 |  |
| E158 |  | E128 |  |
| E159 |  | 26 | Qujing (5), Yunnan |
| E160 |  | 28 |  |
| E170 |  | 31 |  |
| E171 |  | E34 |  |
| E172 |  | E39 |  |
| E173 |  | E108 | Yuxi (3), Yunnan |
| E173-1 |  | E108-1 |  |
| E174 |  | E108-2 |  |
| E181 |  |  |  |
| E182 |  |  |  |
| E230-1 |  |  |  |
| E232-1 |  |  |  |
| E252 |  |  |  |
